# Field Performance of Open-Ended Prestressed High-Strength Concrete Pipe Piles Jacked into Clay

**DOI:** 10.3390/s18124216

**Published:** 2018-12-01

**Authors:** Hai-Lei Kou, Wen-Zhou Diao, Tao Liu, Dan-Liang Yang, Suksun Horpibulsuk

**Affiliations:** 1 College of Engineering, Ocean University of China, Qingdao 266100, China; dwzouc@163.com (W.-Z.D.); danliang1995@163.com (D.-L.Y.); 2 Cooperative Innovation Center of Engineering Construction and Safety in Shandong Blue Economic Zone, Qingdao 266033, China; 3 College of Environmental Science and Engineering, Ocean University of China, Qingdao 266100, China; ltmilan@ouc.edu.cn; 4 School of Civil Engineering, and Center of Excellence in Innovation for Sustainable Infrastructure Development, Suranaree University of Technology, Nakhon Ratchasima 30000, Thailand; suksun@g.sut.ac.th

**Keywords:** open-ended pipe piles, field test, static loading test, soil plugging, residual forces

## Abstract

The behavior of open-ended pipe piles is different from that of closed-ended pipe piles due to the soil plugging effect. In this study, a series of field tests were conducted to investigate the behavior of open-ended prestressed high-strength concrete (PHC) pipe piles installed into clay. Two open-ended PHC pipe piles were instrumented with Fiber Bragg Grating (FBG) sensors and jacked into clay for subsequent static loading tests. Soil plug length of the test piles was continuously measured during installation, allowing for calculation of the incremental filling ratio. The recorded data in static loading test were reported and analyzed. The distribution of residual forces after installation and the effect on the bearing capacity were also discussed in detail. The test piles were observed to be in partially plugged condition during installation. The measured ultimate shaft resistance and total resistance of the test piles were 639 and 1180 kN, respectively. The residual forces locked in the test piles after installation significantly affected the evaluation of the axial forces, and thus the shaft and end resistances. It tended to underestimate the end resistances and overestimate the shaft resistances if the residual forces were not considered in the loading test. However, the residual forces did not affect the total bearing capacity of open-ended PHC pipe piles in this study.

## 1. Introduction

Prestressed high-strength concrete (PHC) pipe piles are commonly adopted as deep foundations in the coastal areas of Southeast China. The installation methods usually involve jacking and driving. A vibration and predrilling method is also utilized in the field. Compared with other installation methods, the jacking method is preferred as it avoids noise, vibration and slurry pollution. Hence, it is more suitable to install piles in urban areas. The performance of jacked pipe piles during and after installation has becomes a focus of attention in recent years [1,2], but many issues remain to be solved, in particular for open-ended pipe piles, such as the termination criteria, the bearing capacity, the design methods and so on [3,4]. This is mainly because the physical properties involved in open-ended pipe piles are extremely complicated [5,6].

Many references have reported on the behavior of jacked steel H-piles, including the effect of excess pore water pressure, the set-up, the estimation of shaft and end resistances and so on [7]. As full displacement piles, the steel H-piles have no soil plug effect during installation [8,9]. However, for non-displacement or partial displacement piles, soil plugging was identified as an important factor in pile behavior [10,11]. A number of field and model tests were conducted to study the effect of soil plugging on the behaviour of open-ended piles jacked into sand [12]. Kou et al. [13,14] revealed that the loading-settlement behavior was closely related to the development of soil plugs. Yu and Yang [15] compared major open-ended pipe pile design methods and proposed the Hong Kong University (HKU) method to estimate the end bearing capacity of open-ended steel piles in sand based on the Imperial College Pile (ICP) and the University of Western Australia (UWA) methods [16,17]. The proposed HKU method was capable of producing satisfactory predictions over a wide range of pile lengths, diameters and slenderness. Moormann et al. [18] adopted Coupled Eulerian-Lagrangian (CEL) method to simulate the soil plug behavior in open-ended steel piles in loose sand to estimate the current design methods. For silty clay, soil plug behavior research focuses on open-ended steel piles with model tests [19] or field tests [20,21]. Few studies were conducted to investigate the soil plugging effect to the performance of jacked open-ended concrete pipe piles. Liu et al. [22] investigated the formation of soil plug in open-ended concrete pipe piles in the field, however, the effect of soil plugging on pile behavior was not discussed.

Further, the conventional strain gauges used in the monitoring of axial strains has some disadvantages [23]. Fiber optic sensors are becoming a well-established technology for a variety of geophysical and civil engineering applications [24]. Schmidt-Hattenberger et al. [25] conducted strain measurements by fiber Bragg grating sensors for in situ pile loading tests. The monitored strain results of fiber Bragg grating sensors agree fairly well with conventional concrete strain gages. Authors have also examined the skin friction between piles and subsoils as well as the settlement behavior of the pile based on the measured strain data. Klar et al. [26] conducted a static loading test on bored piles based on fiber optic technology and discussed the differences between fiber measurements and conventional discrete measurements. Doherty et al. [27] used a fiber sensing system to monitor the axial loads of soil-cement injected precase piles and steel pipe piles in the field. The advantages of fiber optic sensors compared to conventional strain gauges are long-term performance, resistance to corrosive environments, immunity against electromagnetic interferences, array-capability by wavelength-demultiplexing, and miniaturized size. Hence, there is a practical demand to investigate the behaviour of open-ended concrete pipe piles jacked into silty clay considering soil plugging using novel fiber optic sensors [28].

In this study, Fiber Bragg Grating (FBG) sensors were adopted to instrument two open-ended PHC pipe piles to study the soil plugging effect on pile behaviour when jacked into clay. The performance of test piles was continually monitored during the installation and the loading tests. The main objectives of this study were to investigate: (1) the behaviour of open-ended PHC pipe piles jacked into clay; (2) the soil plugging during and after jacking; (3) the residual forces after installation and the effect to the evaluation of total bearing capacity; (4) the loading transfer and settlement mechanisms of the test piles in loading test.

## 2. Site Conditions and Test Program

### 2.1. Site Description

**Figure 1:**
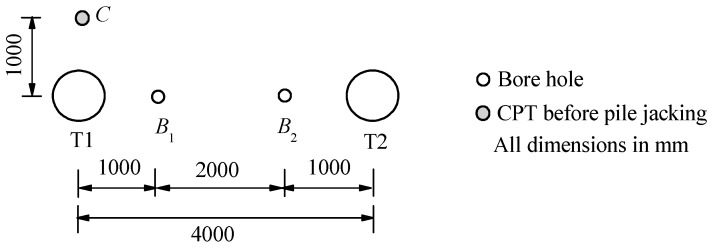
In-situ test layout.

**Figure 2:**
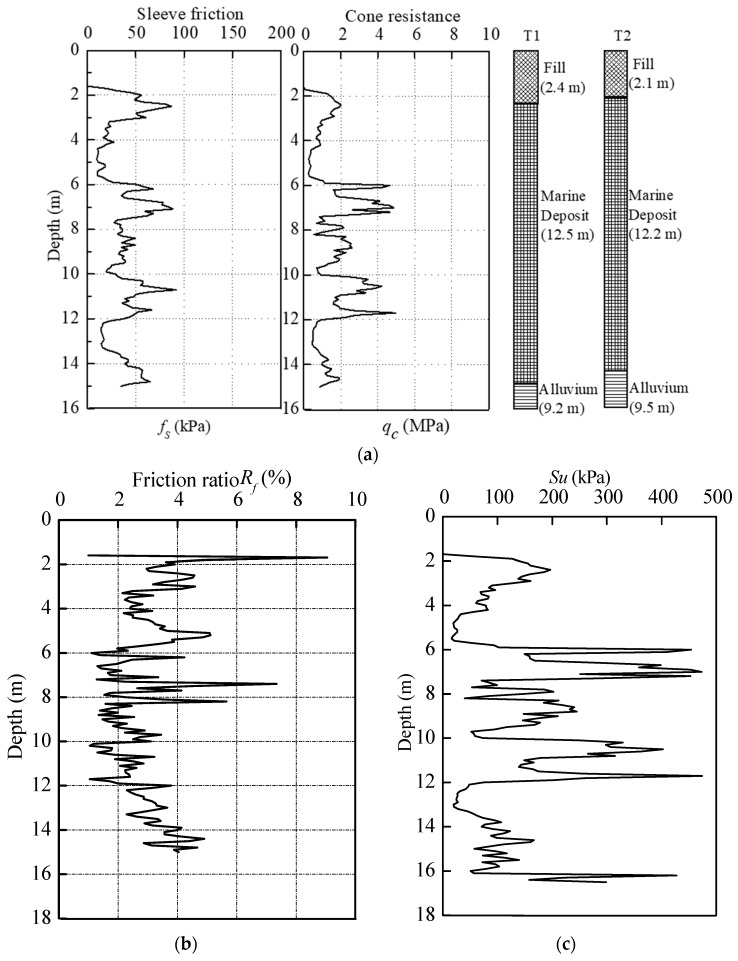
CPT data and Su values in test site. (**a**) Soils profiles and CPT values along depth; (**b**) Friction ratio with depth; (**c**) Undrain shear strength with depth.

Full-scale tests were conducted at a site in Hangzhou, China. Before the test pile installation, two auger borings *B*_1_ and *B*_2_, and one cone penetration test (CPT) were conducted to investigate the soil information, as shown in Figure 1. Figure 2a,b show the soil profiles from boreholes with the cone resistance *q_c_*, sleeve friction *f_s_* and friction ratio *R_f_*. It is indicated that the test site is in sequence by 2.1 to 2.4 m of fill layer, 2.1 m of silty clay, 3.5 m of sandy clay, 2.0 m of muddy-silty clay, 2.5 m of silty clay, and 9.2 to 9.5 m thick alluvium layer. The soil properties in embedded length of test piles are summarized in Table 1. 

The coefficient of compressibility *α*_1-2_, defined as the decrease in volume per unit volume produced by a unit change of pressure, given in the table was determined through one-dimensional compressibility test. The cohesion *c* and friction angle *φ* were determined using direct shear test. The undrained shear strength *S_u_* is calculated from CPT results according to Equation (1) proposed by Schmertmann [29]:

*S_u_* = (*q_t_* − *σ_v0_*)/*N_kt_* (1)

where *q_t_* is the modified cone resistance; *σ_v0_* is the total overburden pressure; *N_kt_* is a cone bearing capacity factor. A *N_kt_* value of 10 was used here.

### 2.2. Test Piles Details and Instrumentation

In order to separate all resistance components for partially plugged open-ended piles, the instrumented double-walled pile system is usually used in model tests [30,31] or full-scale field tests [32]. In this paper, the FBG sensors are installed along test piles using embedded methods. However, the survival rate of embedded FBG system in pile instrumentation is lower than that of conventional sensors because the exposed grating points are very fragile and require further cable protection [33]. Hence, an epoxy resin was used in this test to seal the FBG sensors and cables.

The test piles used in this study are two open-ended PHC pipe piles with 400 mm outer diameter, 75 mm wall thickness, and 13.0 m length. Details are given in Table 2. 

**Figure 3:**
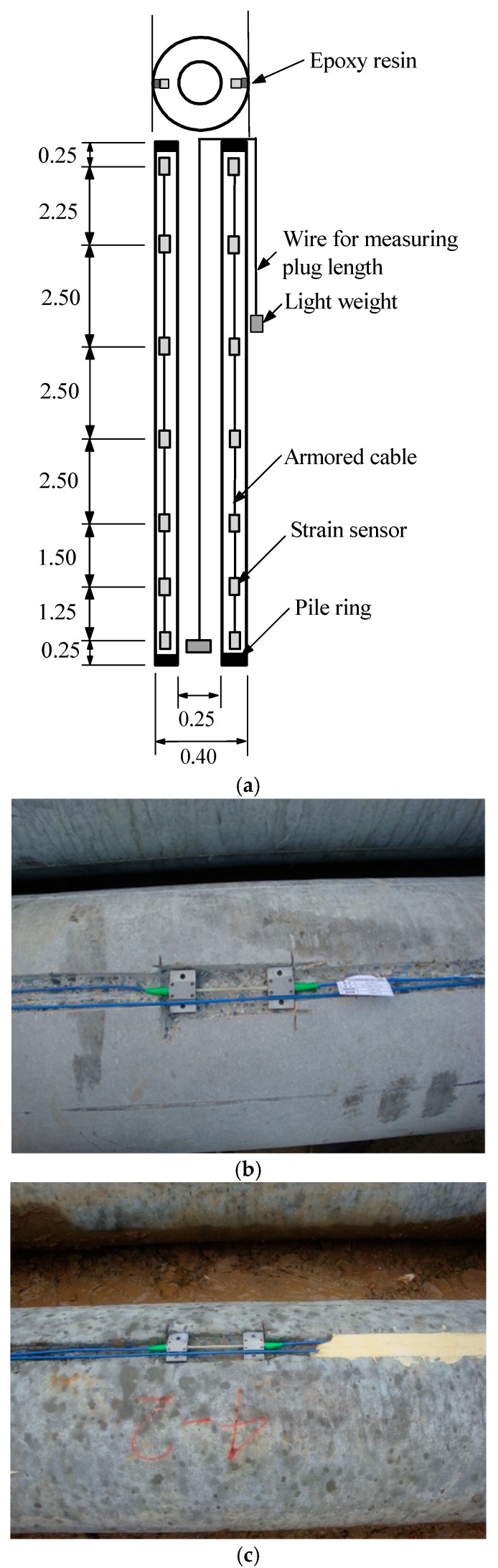
Strain gauges installation on test piles. (**a**) Schematic arrangement for strain gauges (Unit: m); (**b**) Installation of strain gauges along test piles; (**c**) Strain gauges sealed with epoxy resin in field.

Fourteen FBG sensors were installed in each test pile at seven levels to monitor the axial strain along the test piles and then separate the end and shaft resistances. The detailed configuration of FBG sensors instrumentation is shown in Figure 3a. The sensors were installed about 2.5 m intervals and closer together near the pile toe. After two shallow grooves with 50 mm width and 15 mm depth were created, all FBG sensors were attached on the groove bottom according to pre-designed intervals. Then the FBG sensors were sealed with epoxy resin to prevent any damage caused by groundwater and the surrounding soils during jacking, as shown in Figure 3b,c. The axial strain recorded by FBG sensors could be transformed into axial forces. The Young’s modulus of the test piles material was taken as 3.6 × 10^4^ N/mm^2^ in this study without considering pre-stressing. The end resistance was estimated with linear extrapolation method using the measured data. As discussed above, the shaft resistances between soil plug and inner surface were not measured in this program. In other words, the estimated end resistances include annulus and plug resistances.

### 2.3. Test Program

**Figure 4:**
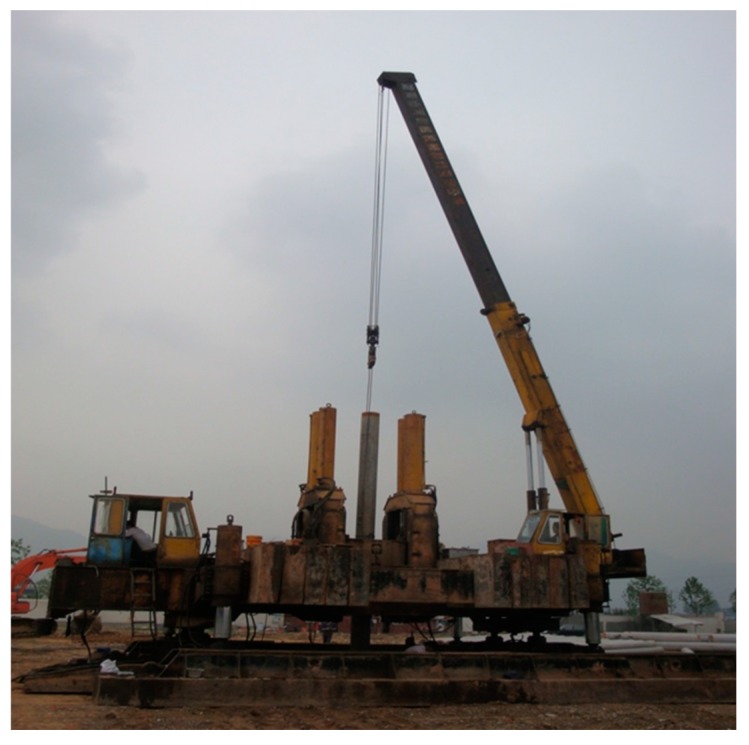
Jacking machine used in the test.

Test piles were installed using a ZYJ680A jacking machine (Figure 4), which has a loading capacity of 6.8 tons. The jacking machine includes two components of clamps and hydraulic jacks. In jacking, the clamp with four arc pieces was used to stabilize against the pile vertically. Then a hydraulic jacking force was applied to push the piles. After the hydraulic clamp reached the maximum travel range, the hydraulic clamp was released and moved up to the top position. The step was repeated to push the piles down until the test piles were installed into the ground. Two test piles were both jacked into 13.0 m depth and founded in the layer of marine deposits. The ultimate bearing capacity of the test piles was estimated as 1200 kN from the calculation before loading test.

**Figure 5:**
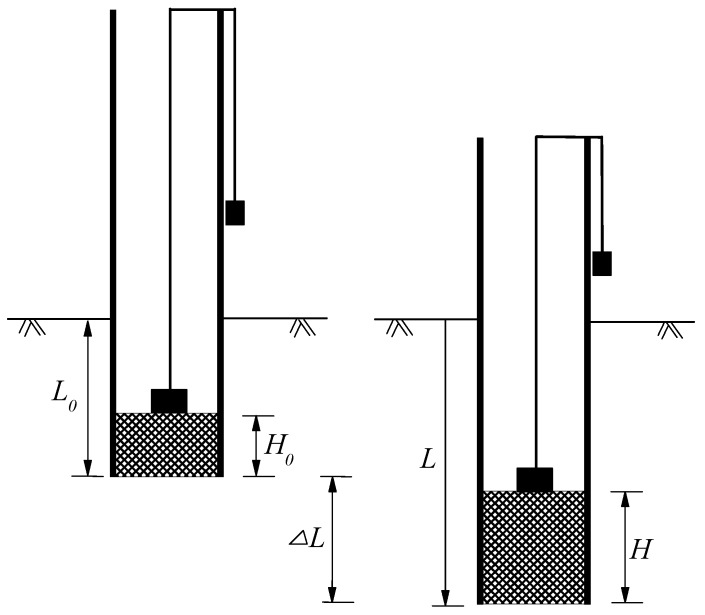
Measurement of soil plug length during pile jacking.

The jacking forces were recorded to assess the drivability of the test piles. The soil plug length during jacking was continuously measured using two different weights, as shown in Figure 5. 

**Figure 6:**
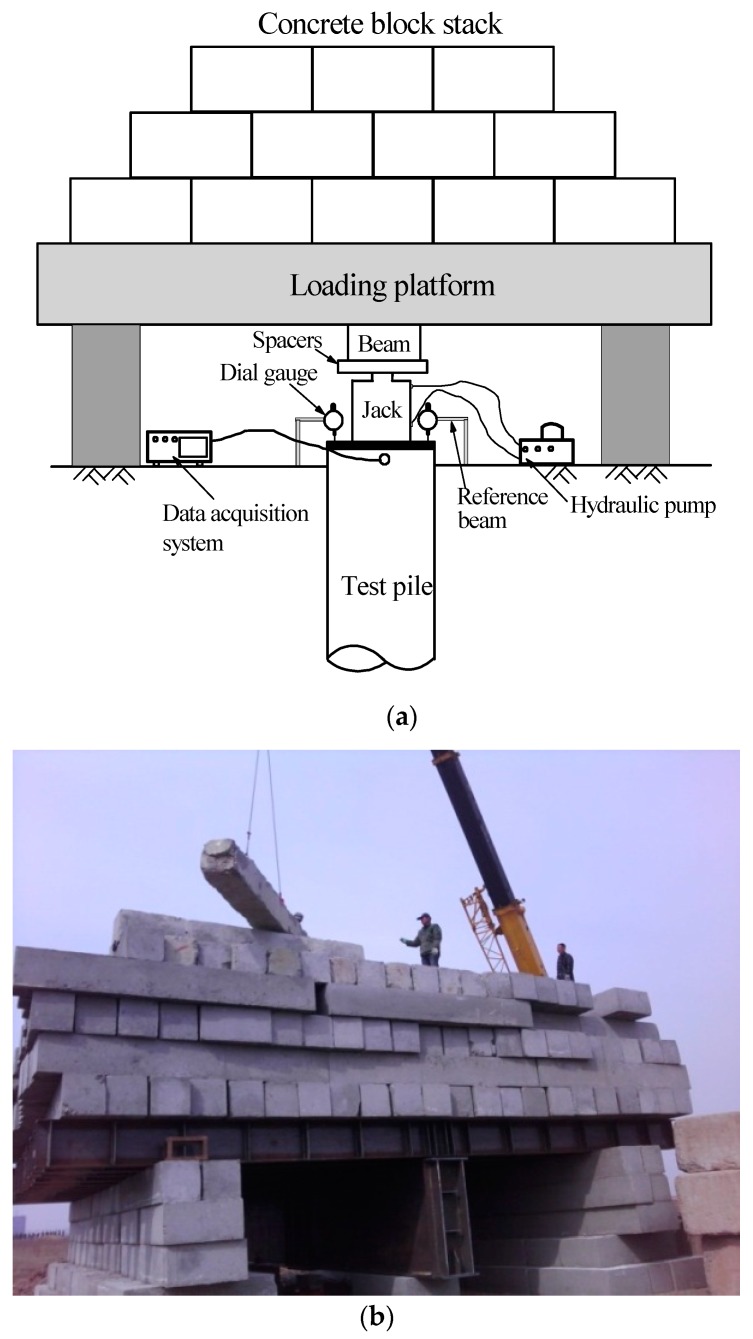
Static loading test of T2. (**a**) Schematic view; (**b**) Static loading test in field.

The loading test was conducted on test piles of T2 at 17 days after jacking, as illustrated in Figure 6. The total applied loads to the piles head were controlled by a hydraulic pump. The vertical displacement was monitored using two dial gauges mounted in the reference beams. The values of all FBG sensors attached to T2 were set to be zero before loading in order to measure the axial forces induced by applied loads. The static loading test was slowly maintained loading test and conducted in accordance with the Chinese technical code for testing of building foundation piles [34], with no unload-load loops.

## 3. Test Resultis and Discussion

### 3.1. End and Shaft Resistance during Jacking

**Figure 7:**
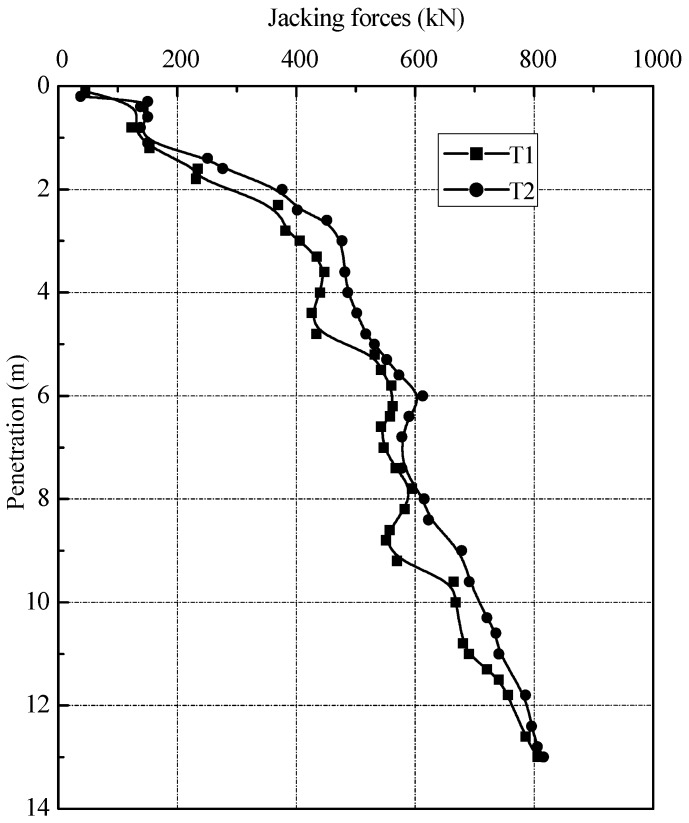
Jacking forces versus penetration depth.

**Figure 8:**
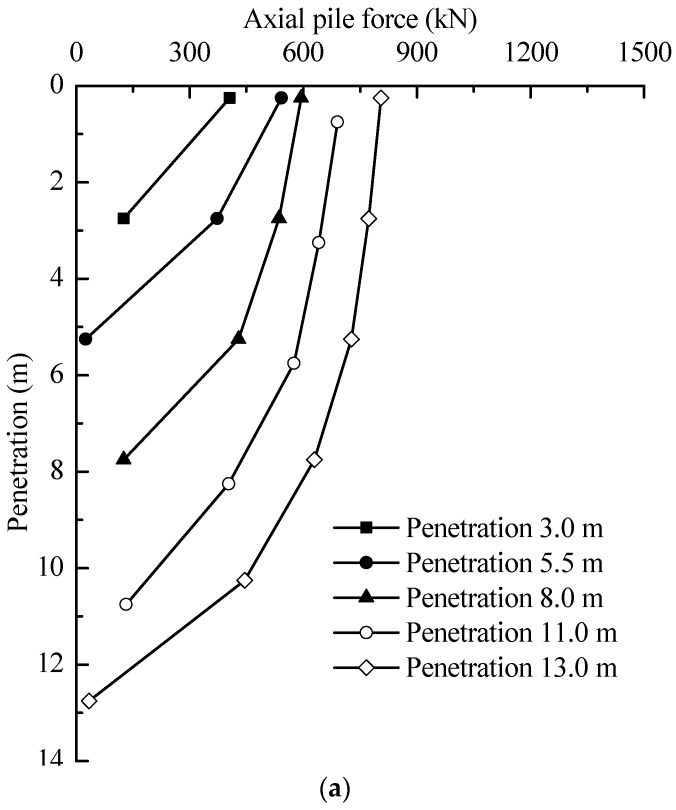

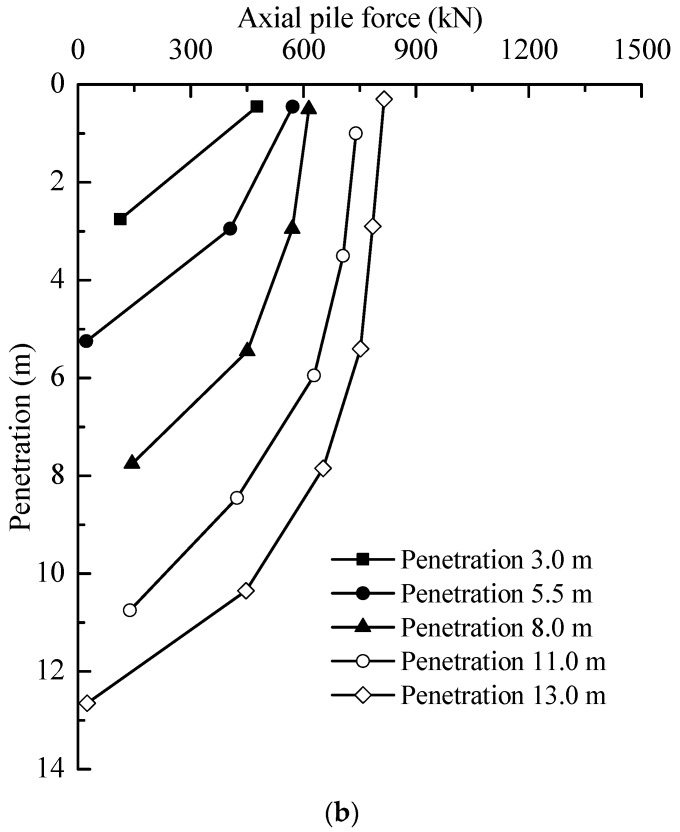
Variation of axial pile forces during jacking: (**a**) T1; and (**b**) T2.

The jacking forces of test piles during installation are plotted in Figure 7. It can be seen from the figure that the jacking forces increased with penetration depth. At final depth of 13.0 m, the jacking forces were 806 and 815 kN for T1 and T2, respectively. The induced axial forces along test piles are illustrated in Figure 8. It is indicated that the axial forces increased with jacking depth and the distribution shown similar characteristics to each other. It also should be noticed that the induced axial forces in Figure 8 are different from the jacking forces in Figure 7. The induced axial force on the piles head is the same as the jacking forces at the corresponding depth in Figure 7.

**Figure 9:**
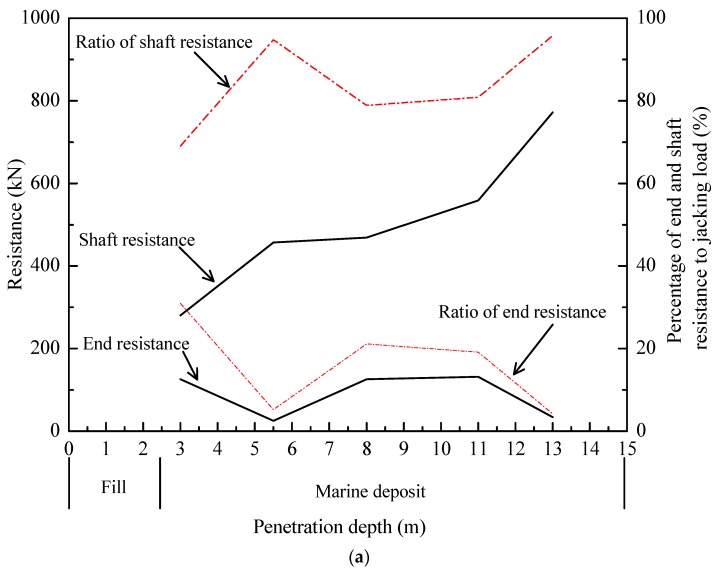

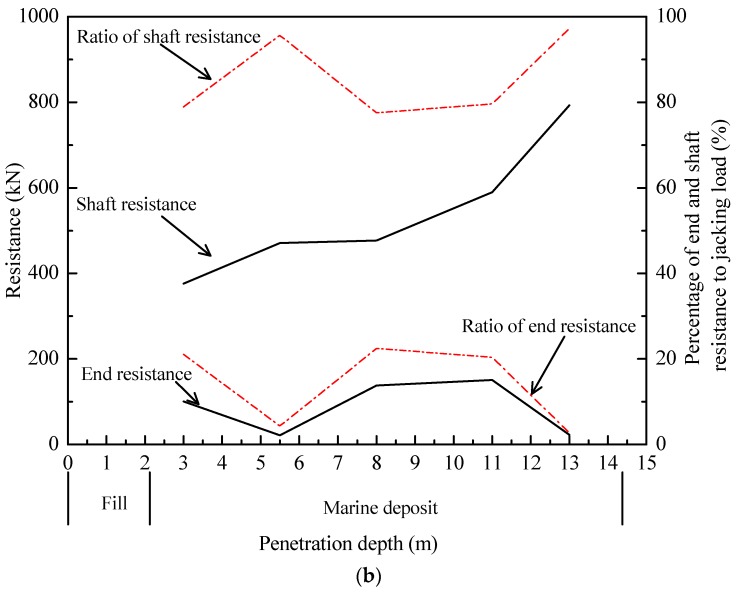
Variation of end and shaft resistance during jacking: (**a**) T1; and (**b**) T2.

For a better view of the loading transfer, the end and shaft resistances are separated and shown in Figure 9. 

**Figure 10:**
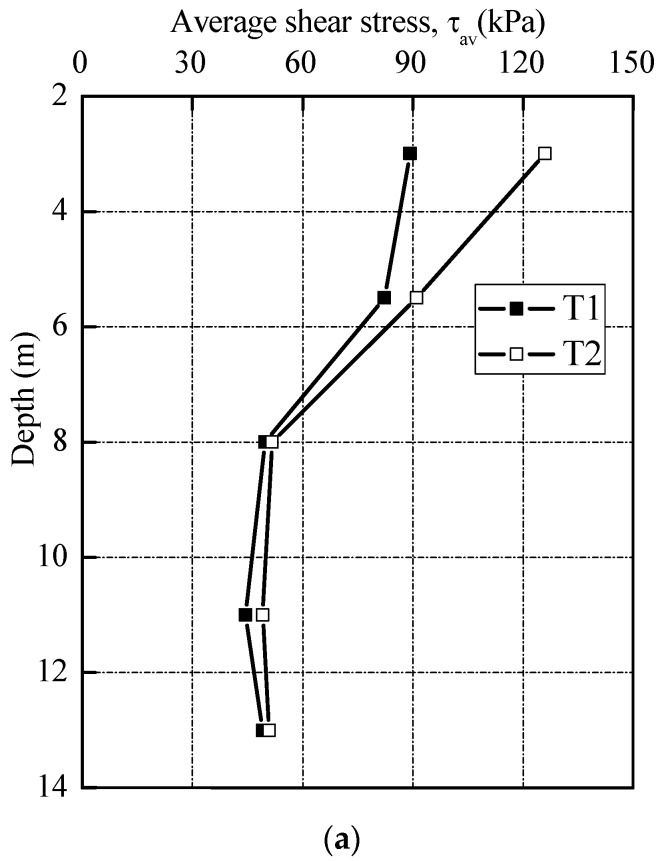

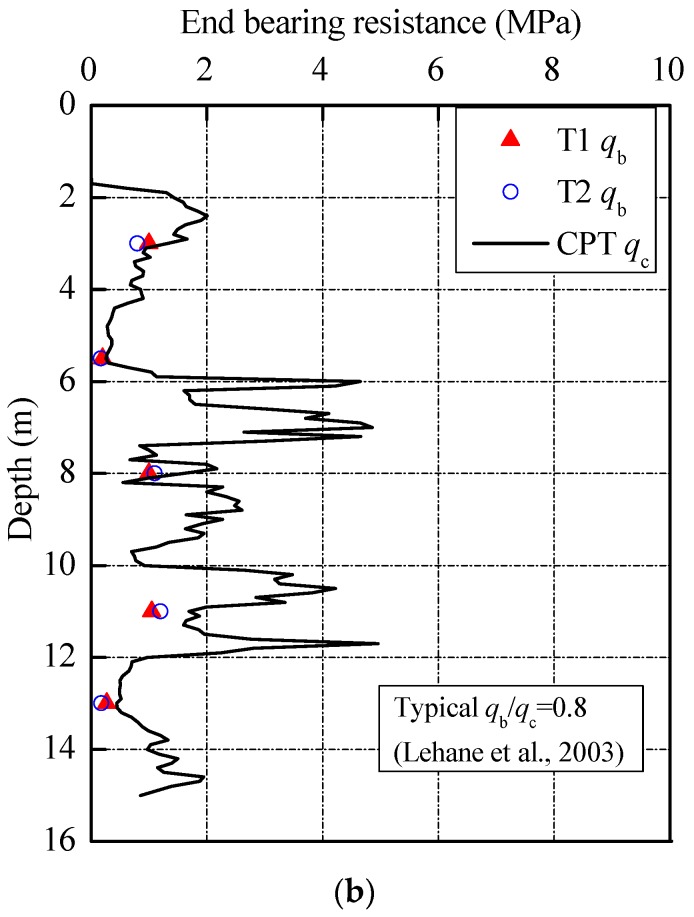
Pile installation stresses with depth. (**a**) Average shear stresses profile; (**b**) Average end bearing stress.

The shaft resistances of T1 and T2 consistently increased with installation, while the end resistances were in a range from 21 to 151 kN. At a penetration depth of 5.5 m, the shaft resistances of test piles reached a level of 457.3 and 471.2 kN, taking on 94.8 and 95.7% of the jacking forces, respectively. At 13.0 m, the shaft resistance for T1 was about 771.6 kN, amounting to 95.8% of jacking forces. For T2, the shaft resistance reached a value of 793.1 kN, while the end resistance was about 22.7 kN. The corresponding ratios of the shaft and end resistances to the jacking forces were 97.2 and 2.7%, respectively. The average shear stresses (*τ*_av_ = shaft load/shaft area) and unit end resistance (*q*_b_) measured during installation are plotted against depth, as shown in Figure 10. 

The *q*_b_ is the force per unit cross section area, which the cross-section area is calculated as a closed-ended pipe piles. In order to compare with the cone resistance *q*_c_ from CPT, the *q*_c_ values at corresponding depths were also marked in the figure. It is apparent that these *q*_b_ values are around 0.8 *q*_c_, which is in good agreement with those reported in previous database [35,36].

### 3.2. Soil Plug Behavior

Soil plugging plays an important role in controlling the behavior of open-ended pipe piles during installation and loading tests. The degree of soil plugging can be represented by plug length ratio (PLR) and incremental filling ratio (IFR), defined as:

PLR = *H*/*L* (2)

IFR = *dH*/*dL* × 100(%) (3)

where H/L is the length of soil plug H with reference to an installation length of piles L; dH/dL expresses the increase of soil plug length H per unit increase of installation depth L.

**Figure 11:**
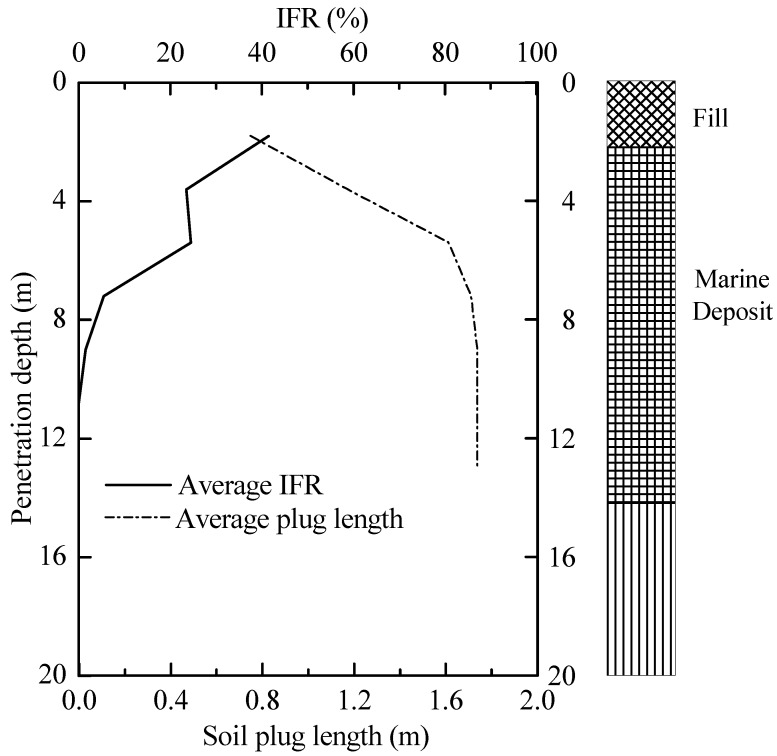
IFR and soil plug length versus penetration depth for test piles.

During installation, the soil plug condition can be divided into three categories: fully coring (IFR = 100%), fully plugged (IFR = 0) and partially plugged (0 < IFR < 100%). The variations of average soil plug length and IFR with installation are illustrated in Figure 11. The test piles were partially plugged from the outset of pile installation. The IFR decreased sharply from 23.2% to 5.3% at the depth of 6.0 m and then decreased to near zero at penetration depth of 11.0 m. The abrupt change of IFR is due to the existence of a relatively stiff interlayer that is verified by the CPT-*q*_c_ trace in Figure 2a. The PLR values of T1 and T2 recorded at the end of installation were 0.13 and 0.14, respectively. After the loading test, there was no change of the measured soil plug length. This reinforces the fact that soil plug behaviour of open-ended pipe piles is very different during installation and static loading test.

### 3.3. Residual Forces after Installation

**Figure 12:**
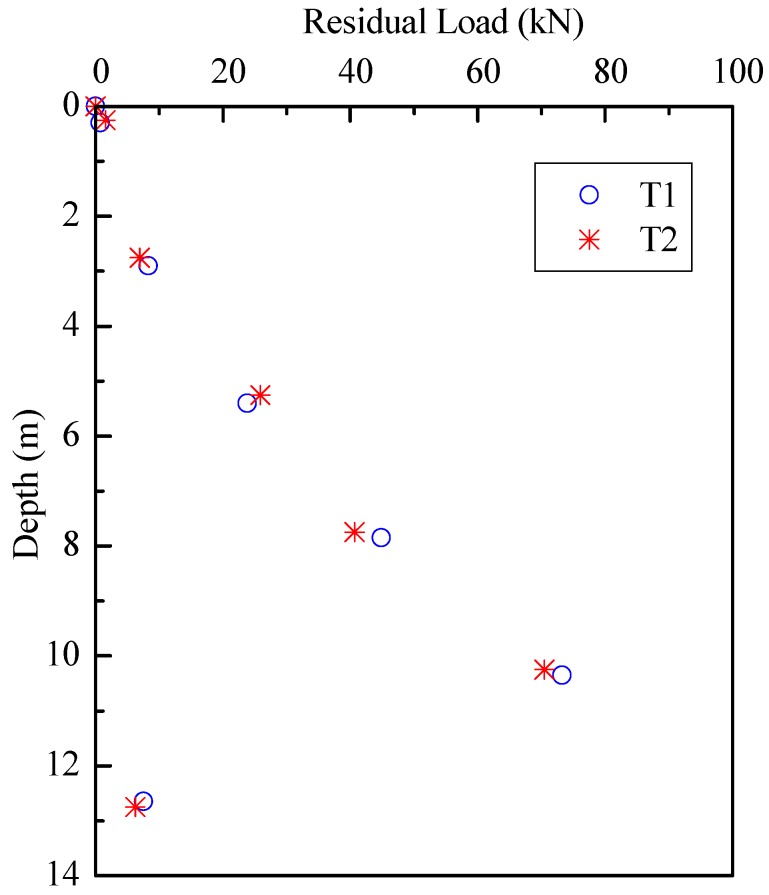
Residual loads distribution after installation.

At the end of each jacking stroke, in particular, after the last jacking stroke, the pile-soil interaction reached a static equilibrium with the recovery of elastic compression caused by compression/tension pulses during jacking. There are still some residual forces locked in the piles and these are always compressive at the pile toe [37]. The magnitude and distribution of locked residual forces in the piles after installation have significant effects on the interpretation of loading transfer, and then end and shaft resistances [38,39]. Through the comparison of FBG sensor readings of pre-installation and post-installation, the residual forces after installation can be determined, as shown in Figure 12. The FBG sensor readings after installation can be recorded several hours after jacking in order to let the pile-soil interaction reached the equilibrium. If the reading was recorded immediately after installation, the residual forces would be overestimated as the piles did not complete the recovery of elastic compression.

Figure 12 illustrates that the residual end forces of T1 and T2 were 9.5 and 8.2 kN, which were 25.2 and 23.8% of the end resistances at 13.0 m, respectively. The residual forces along the inner shaft of the test piles were not measured in this program due to the installation difficulties of FBG sensors on the inner shaft. Therefore, the recorded residual end forces were due to the residual soil plug and residual annulus forces. The magnitude and distribution of the residual forces along piles after installation are significantly affected by pile material, length, cross-sectional area and soil properties [40]. This can explain the similar trend of residual forces in the test piles after installation.

### 3.4. Pile Behavior during Static Loading Test

**Figure 13:**
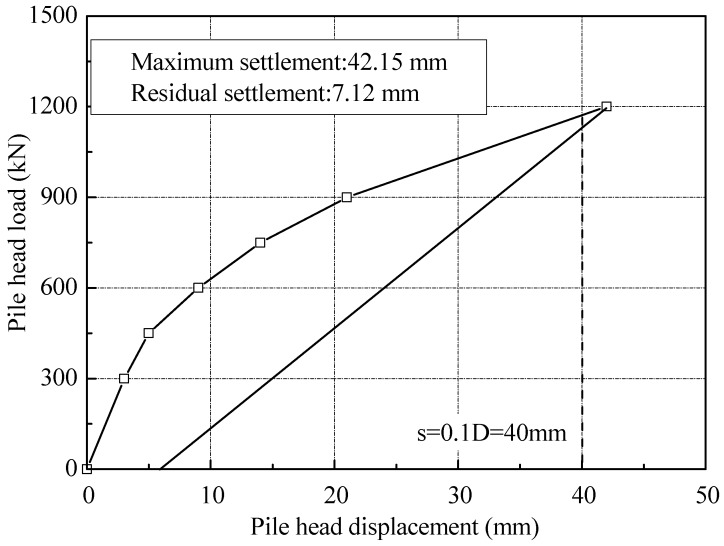
Load-settlement curve for static loading test of T2.

The load versus settlement curve of T2 measured in the static loading test is drawn in Figure 13. At the loading of 600 kN, the settlement of piles head was 9.21 mm, about 2.3% of pile diameter. While at the maximum loading of 1200 kN, the settlement recorded was about 42.15 mm, which was 10.5% of pile diameter. Using the Chinese technical code for testing of building foundation piles [34], the ultimate bearing capacity of the pile T2 was determined to be 1180 kN, which was the load at which the piles settled by 10% of the pile diameter (40 mm). The ultimate end and shaft resistance at the displacement of 40 mm were 541 and 639 kN, respectively. 

**Figure 14:**
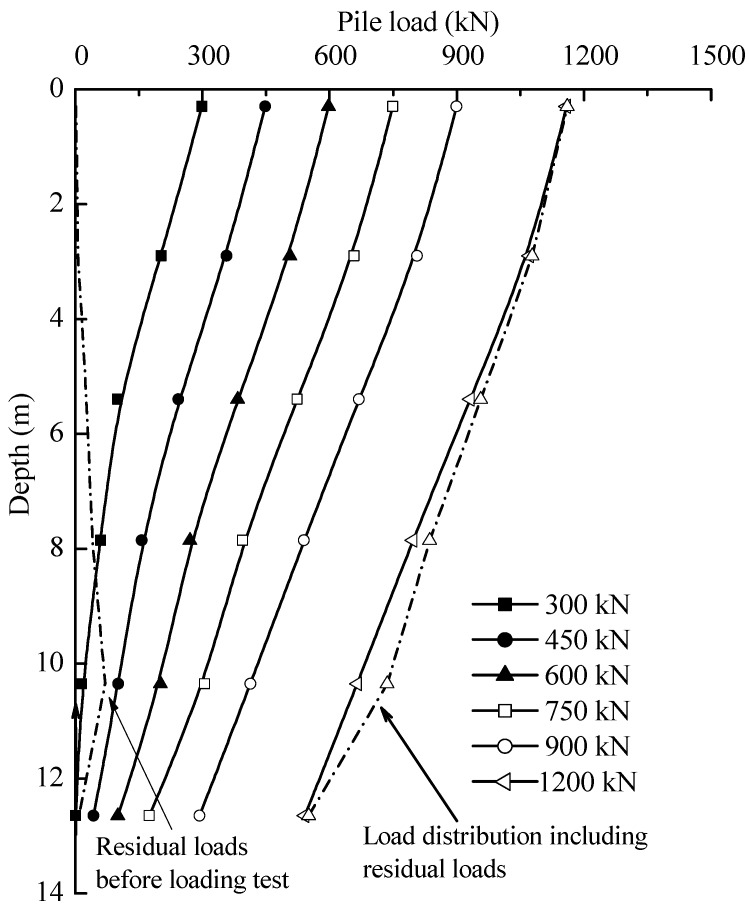
Load distribution curves for test pile.

The axial forces distribution of the test piles T2 in the static loading test is shown in Figure 14. It can be seen that at each load level, a linear reduction of axial forces was observed. It implies that fairly uniform shaft resistance was mobilized in soil layers. It also indicates that the loads applied to the test piles was supported by shaft resistances in initial loading. The applied loads were then gradually transferred to the pile end.

**Figure 15:**
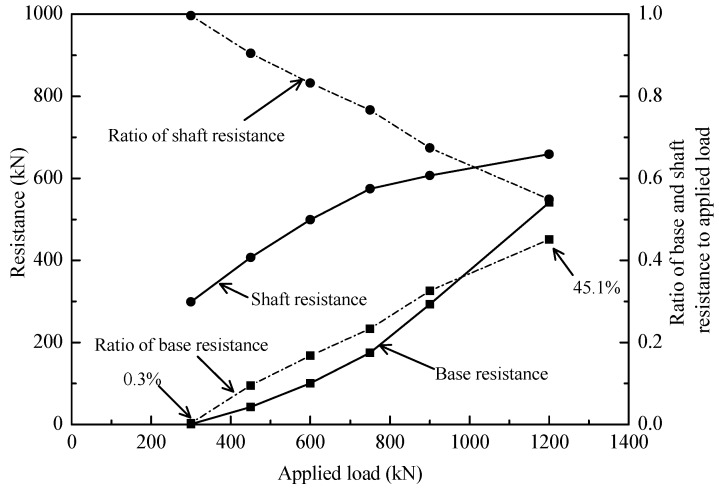
Variation of base and shaft resistance during loading test.

The residual forces before loading test and the final load distribution including residual forces are plotted as dotted lines in Figure 15. The influence of residual forces appeared not to be negligible on the loading transfer in the static loading test. The end resistances of test pile T2 will be underestimated by 4.2% if not considering the residual forces in static loading test. For equilibrium to be established, the upward residual end forces must equal the downward resultant of the residual shaft forces. As such, the shaft resistances will be overestimated in the interpretation of pile bearing capacity if the residual forces are not taken into account. The test results are consistent with the findings of other researchers, e.g., [41,42]. As the aim of a loading test is to estimate the total bearing capacity, residual forces should not be considered as the summation of residual shaft and end forces for the pile must equal zero. However, we should account for residual forces if the goal of the loading test is to assess the end and shaft resistances for design.

**Figure 16:**
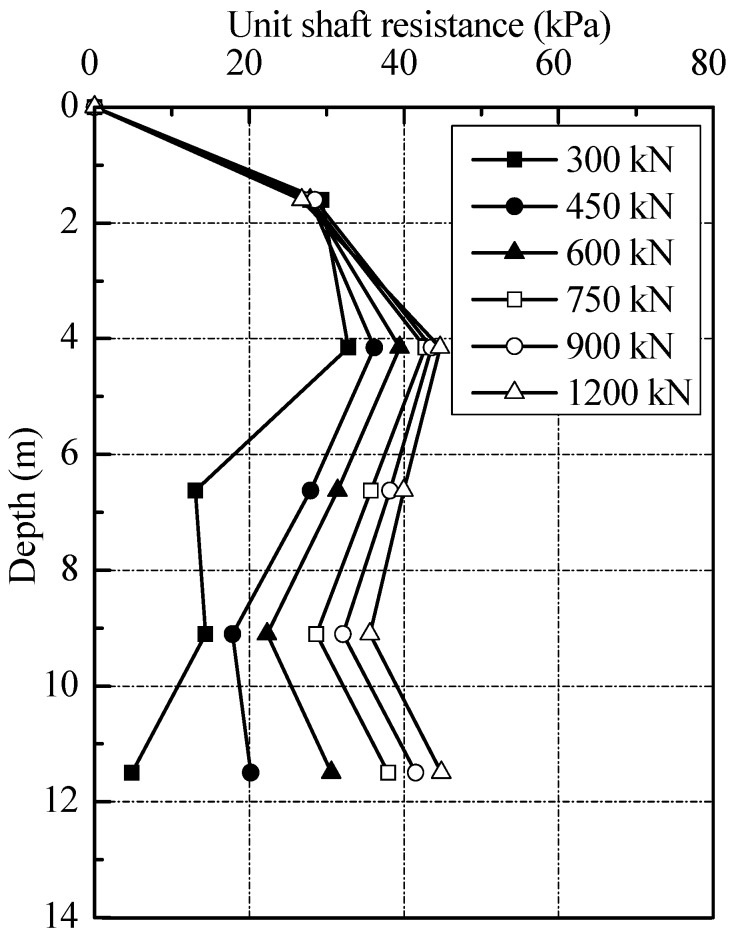
Distribution of unit shaft resistance of test pile.

To enable a better assessment, Figure 15 includes the end and shaft resistances (without residual forces) at each loading level, together with the values of the ratio of the end resistances to applied loads. The ratio of end resistances to applied loads of 300 kN was 0.3%; it was to 45.1% with the applied load of 1200 kN. The results indicated that most of the applied loads were undertaken by shaft resistances rather than end resistances in loading test. The distribution of unit shaft resistance can be deduced from Figure 15, as shown in Figure 16. It can be seen that the unit shaft resistance was gradually mobilized as the loads increased. The relative displacement δ*i* between soil layer *i* and pile during static loading test can be expressed by:

(4) $$\delta_{i}\;=\;S-\;\sum_{j=1}^{i}\frac{L_{i}}{2}\left(\varepsilon_{j}+\varepsilon_{j+1}\right)$$

where *S* is the pile head settlement at different loading;$\;\varepsilon_{j}$ is the pile strain at *j* level; and *L_i_* is the length of the pile located at *i* level.

**Figure 17:**
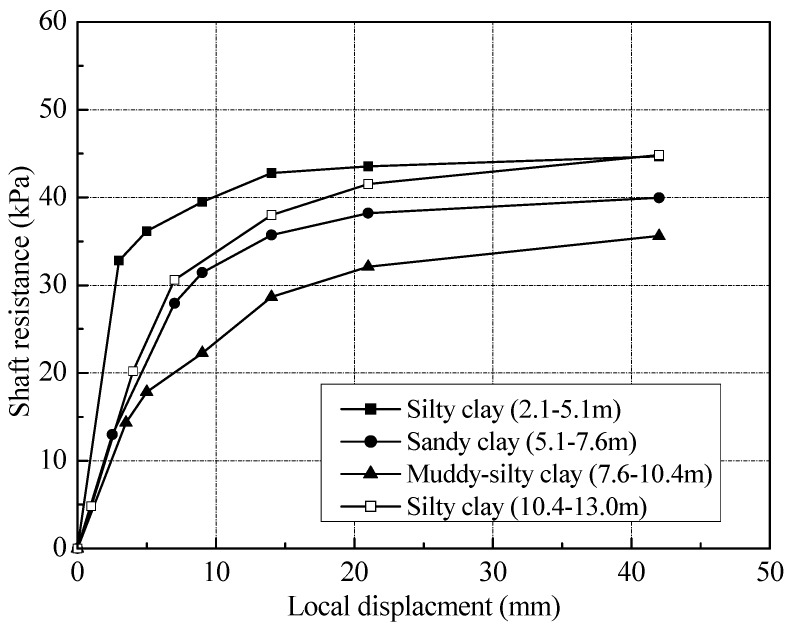
Local shaft resistance versus local displacement of test pile.

Figure 17 shows the relationship between local shaft resistance and local displacement at various depths for T2. The unit shaft resistance in Figure 16 and Figure 17 does not include the residual forces. According to the study of [13,14], the local displacement could be defined as the differences between pile head settlement and the elastic compression of the pile above the depth under consideration, which can be induced from Equation (4). The test results indicated that the local unit shaft resistance had a good correlation with pile-soil relative displacement. At the same layer, the unit shaft resistance increased approximately hyperbolic to the peak value with pile-soil relative displacement. In the silty clay layer, the shaft resistance could be fully mobilized at a relative displacement of 15 mm, about 3.75% of pile diameter. The threshold of slip displacement for full mobilization of the shaft resistance was found to be 13 and 20 mm in sandy clay and muddy-silty clay layers respectively, about 3.25% and 5.0% of pile diameter. Note that the ultimate value of the shaft resistance in silty clay layer was up to 45 kPa. The values in sandy clay and muddy-silty clay layers were about 40 and 35 kPa, respectively. At the end of static loading test, the shaft resistance along the pile has been fully developed.

## 4. Conclusions

This paper described the results of a full-scale test on open-ended PHC pipe piles instrumented with FBG sensors in clay. The behavior of test piles was discussed during jacking and static loading test. The main conclusions could be obtained as follows:

(1) The FBG sensoring technology was proved be feasible to measure the axial forces of jacked open-ended PHC pipe piles in clay. It is revealed that the axial forces along PHC pipe piles can be obtained through FBG sensor multiplexing technology.
(2) The behaviour of open-ended PHC pipe piles is more complicated once the effect of soil plugging is considered. The open-ended PHC pipe piles were jacked into clay in a partially plugged mode while the behaved as fully plugged piles in loading tests. This implies that the soil plugging was very different under installation and static loading conditions.
(3) The residual forces in open-ended PHC pipe piles after installation were large and always were compressive at pile toe. The ratios of residual end forces to end resistances after installation were 25.2 and 23.8%, respectively. The residual forces also significantly affect the interpretation of the load distribution in static loading test. The end resistances of the test piles will be underestimated by 4.2% if the residual forces are not considered. However, the residual forces do not affect the total bearing capacity as the sum of residual shaft and end resistances must equal zero.
(4) Loading test results indicated that the shaft resistance has a good correlation with pile-soil relative displacement. The threshold of slip displacement for fully mobilizing the shaft resistance was found to be 15, 13 and 20 mm for silty clay, sandy clay and muddy-silty clay layers, respectively.

## Figures and Tables

**Table 1:** Subsoil Properties.

Soils Type	Depth (m)	*W* (%)	*γ* (kN/m^3^)	*e*_0_ (%)	*G_s_*	LL (%)	PL (%)	α_1-2_ (MPa^−1^)	*c* (kPa)	*φ* (^o^)
**Silty clay**	2.1~5.1	25.7	19.36	0.73	2.72	30.1	17.9	0.15	14.0	21.5
**Sandy clay**	5.1~7.6	31.2	18.52	0.87	2.69	33.3	26.4	0.18	7.1	29.4
**Muddy-silty clay**	7.6~10.4	44.8	17.07	1.28	2.74	37.0	19.7	0.87	15.8	8.0
**Silty clay**	10.4~13.0	23.4	19.77	0.67	2.72	28.6	15.8	0.22	28.5	22.8

Note: *c* and *φ* were determined using the quick shear tests; *α*_1-2_ was determined using one-dimensional compressibility test.

**Table 2:** Details of Test Piles

Pile number	Size (mm)	Embedded Length (m)	Elastic Modulus (GPa)	Axial Compressive Strength (MPa)
**T1**	400 (75)	13.0	36.0	35.9
**T2**	400 (75)	13.0	36.0	35.9

Note: In size, 400 mm is the outer diameter of the test piles, 75 mm is the wall thickness; 36.0 GPa is the elastic modulus of the test piles material.
